# Supplementary material for: PTWAS: investigating tissue-relevant causal molecular mechanisms of complex traits using probabilistic TWAS analysis
Source: Genome Biol. 2020 Sep 11;21:232. doi: 10.1186/s13059-020-02026-y (PMC7488550; doi:10.1186/s13059-020-02026-y)
Supplement: Supplementary file 1 — Additional file 1 Figure S1: Diagram representing instrumental variable analysis. Figure S2: Distribution of Gini coefficients summarizing the sparsity of the composite IVs in different TWAS scan methods. Figure S3: Allelic heterogeneity in implicated PTWAS signal genes. Figure S4: Heterogeneity of gene-to-trait effects across tissues. Figure S5: Comparison of distributions of colocalization probabilities between all genes and PTWAS significant genes. [file 13059_2020_2026_MOESM1_ESM.pdf]

Additional File 1: Supplementary Figures

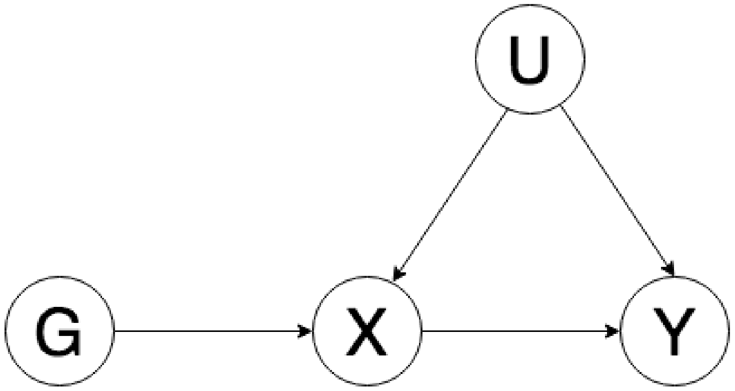

Figure S1: **Diagram representing instrumental variable analysis** Variables G, X, Y, and U represent eQTLs, gene expressions, complex traits and unobserved confounding factors, respectively. The arrow from X to Y represents the causal relationship of interest.

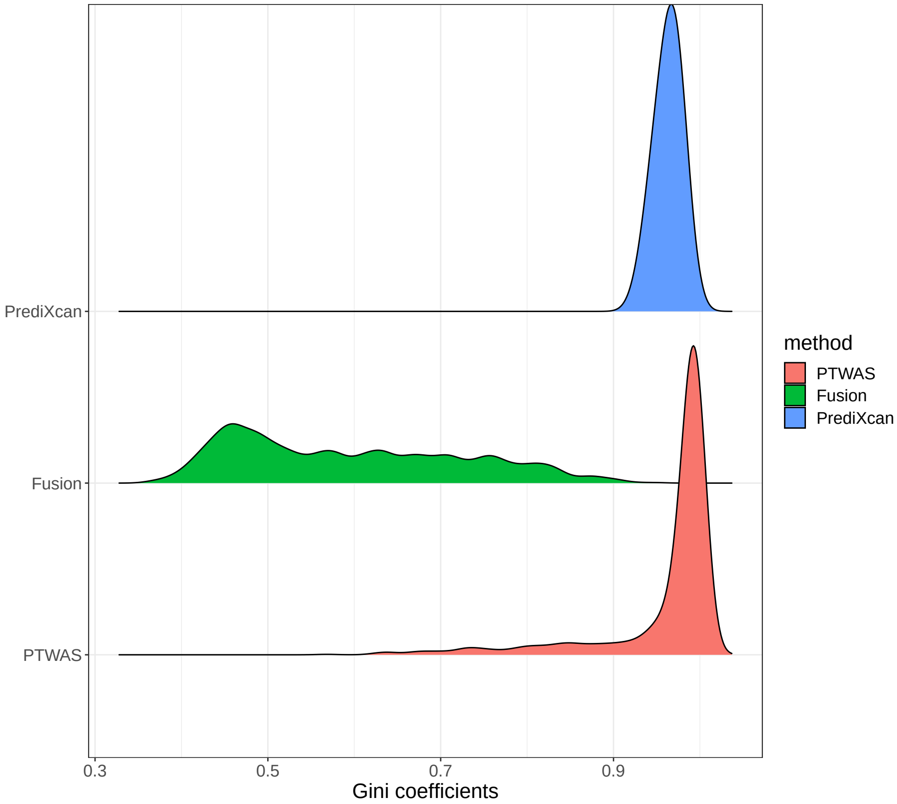

Figure S2: **Distribution of Gini coefficients summarizing the sparsity of the composite IVs in different TWAS scan methods** For each simulated gene, we compute the Gini coefficient based on the assigned weights of all the *cis*-SNPs for computing the composite IV/predicted gene expression levels. A low (i.e.,$\to 0$) Gini coefficient indicates many SNPs play important roles; whereas a high value (i.e.,$\to 1$) suggests only a few SNPs make contributions. Among the methods compared, PrediXcan (based on ElasticNet algorithm) utilizes the most sparse set of SNPs for predicting gene expressions, and TWAS-FUSION utilizes many more SNPs (most of which are likely weak IVs). PTWAS is overall similar to PrediXcan but with a notable long left tail. This is mainly because the weight assignment in PTWAS takes accounts of LD: strong eQTL SNPs that are highly correlated (hence not identifiable) are assigned to comparable weights.

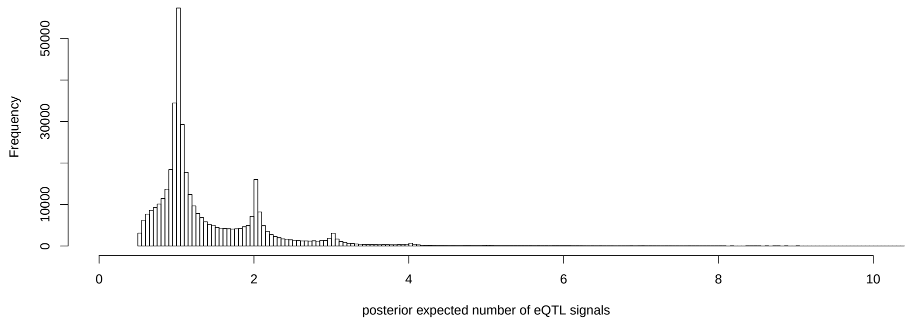

Figure S3: **Allelic heterogeneity in implicated PTWAS signal genes** The histogram shows the distribution of the posterior expected number of *cis*-eQTLs of each unique gene-tissue pair that are implicated in the PTWAS scan and suitable for effect size estimation from the analysis of GTEx data and 114 complex traits. The plot indicates that a substantial proportion of gene-tissue pairs have more than 1 strong eQTLs.

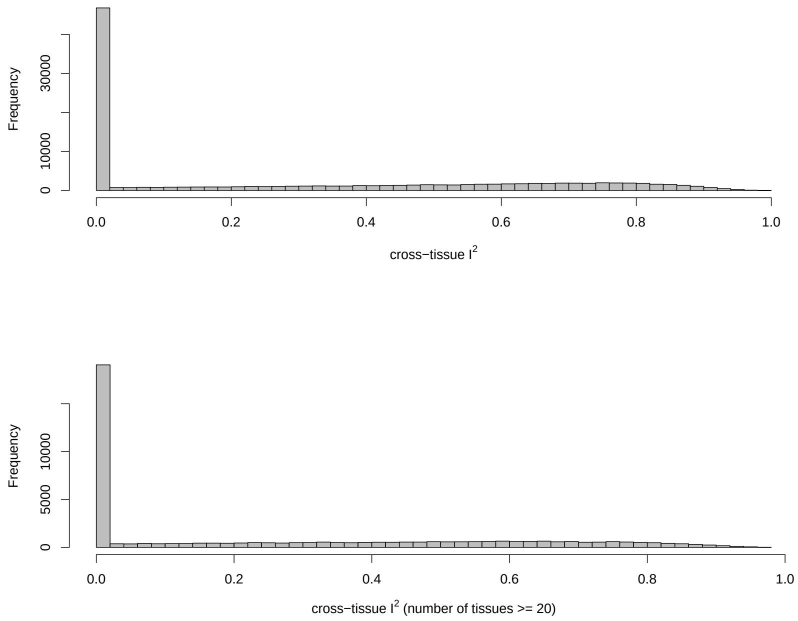

Figure S4: **Heterogeneity of gene-to-trait effects across tissues** The histograms show the distributions of I^2 statistics which quantify the heterogeneity of estimated gene-to-trait effects across different GTEx tissues. The top panel shows all eligible gene-trait paris. The bottom panel shows the gene-trait pairs that are measured in ≥ 20 different GTEx tissues. The overall patterns remain the same.

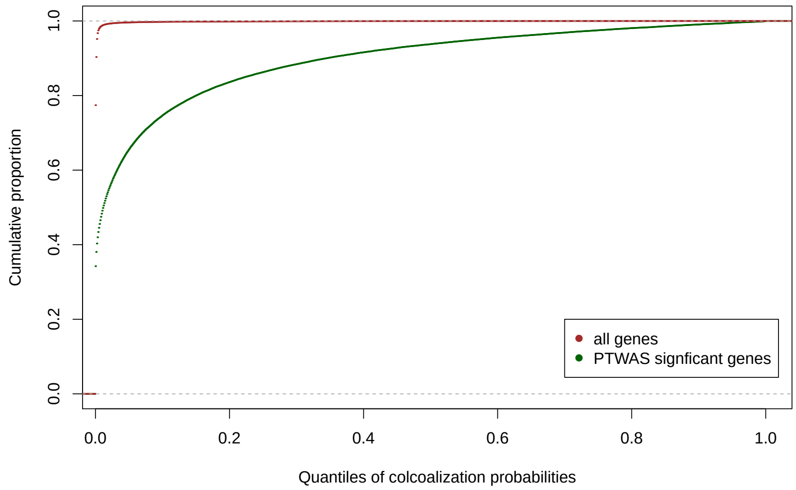

Figure S5: **Comparison of distributions of colocalization probabilities between all genes and PTWAS significant genes** The red line represents the cumulative distribution function (cdf) of colocalization probabilities summarized from all 32,363 candidate genes in all 49 tissues across 114 traits. The green line represents the cdf from the corresponding PTWAS significant genes. It is clear that PTWAS signal genes are enriched with modest to high colocalization probabilities.
