# Supplementary material for: PTWAS: investigating tissue-relevant causal molecular mechanisms of complex traits using probabilistic TWAS analysis
Source: Genome Biol. 2020 Sep 11;21:232. doi: 10.1186/s13059-020-02026-y (PMC7488550; doi:10.1186/s13059-020-02026-y)
Supplement: Supplementary file 7 — Additional file 7 Review history. [file 13059_2020_2026_MOESM7_ESM.docx]

Review History

**First round of review**

**Reviewer 1**

**Were you able to assess all statistics in the manuscript, including the appropriateness of statistical tests used?**

Yes, and I have assessed the statistics in my report.

**Comments to author:**

In the manuscript "Investigating tissue-relevant causal molecular mechanisms of complex traits using probabilistic TWAS analysis", the authors Yuhua Zhang et al, developed a new computation framework by integrating instrumental variables analysis to delineate large scale gene-trait links. Compared with previous methods, this method showed improved power and accuracy for estimation of causal effects. The technique and the identified significant gene-trait pairs could be of broad interest to the field. To be published in Genome Biology, I only have three major points as shown below:

1.     While the authors compared the performance of several strategies using stimulated data, it would be great if the comparison could be expanded to real data, with other newly published methods (i.e. FOCUS) included.

2.     The paper re-discovered several known gene-trait relationships (with citations missing). However, more evidence are needed for validating whether the result is biological meaningful (i.e. the intersection with OMIM database or other approaches as shown in the TWMR paper: https://www.ncbi.nlm.nih.gov/pubmed/31341166)


3.     The related github website needs more detailed tutorials (i.e. installation, parameters) for broad application of the computation strategy. For reference, please check the tutorials of the PediXcan (https://github.com/hakyimlab/PrediXcan/tree/master/Software) or MetaXcan (https://github.com/hakyimlab/MetaXcan). Also it would be great if the computation time and resource could be briefly annotated in the method section.

**Reviewer 2**

**Were you able to assess all statistics in the manuscript, including the appropriateness of statistical tests used?**

Yes, and I have assessed the statistics in my report.

**Comments to author:**

The paper proposes PTWAS, a computational framework to detect association and investigate causal relationships between gene expression and complex traits. The proposed method tries to make use of the established concept and principles from instrumental variables (IV) analysis to claim causal relationships. The idea is interesting and has the potential to play an important role in understanding the complex causal mechanism of a biological system. However, I have the following concerns about the current form of the paper.

(1)     The logic flow and the work flow of the proposed method need to be better demonstrated and explained. The proposed method is a complicated data processing procedure involving multiple inputs and many steps. The paper, however, does not put together all these ingredients into a well-organized system whose logic can be easily followed. The work flow showed in Figure 1 is somehow too "high-level" with many key components and key steps missing, and thus does not help much for understanding the proposed approach in depth. It is not clear to me what are the inputs (in a concrete form) of the proposed approach and what are the intermedias obtained during the learning process. I hope the authors can better the work flow and highlight the inputs, key intermedias and final outputs in a concrete form.
(2)     It's not transparent how the causal assumptions are verified. Verifying the causal assumptions is a critical step for the proposed method. Based on the statement of the paper, this task heavily depends on the "two-sample design". However, the paper talks very little about the design, how data are used based on the design, how and why such a design can help establish a proper IV, leaving a significant gap in understanding.
(3)     Some notations in the paper are not explicitly defined or explained. For example, the concrete meanings of  in P12-L60 and  in P13-L7 are missing;  in page 13 are not defined. These vague notations and concepts result in confusions and obstacles in reading and understanding.
(4)     Result validation and comparison. The paper compares PTWS to competing methods such as PrediXcan, TWAS-Fusion, SMR via simulation, and compares PTWS to colocalization analysis on real data. I am wondering whether PTWS can also be compared to PrediXcan, TWAS-Fusion, SMR on real data. Moreover, the paper chooses the "colocalization probability" as the standard for result validation. Why colocalization probability is a proper choice? Do we have some other measurements can serve better for the validation purpose?

**Authors Response**

**Point-by-point responses to the reviewers’ comments:**

Reviewer 1

In the manuscript “Investigating tissue-relevant causal molecular mechanisms of complex traits using probabilistic TWAS analysis”, the authors Yuhua Zhang et al, developed a new computa­tion framework by integrating instrumental variables analysis to delineate large scale gene-trait links. Compared with previous methods, the new method showed improved power and accuracy for estimation of causal effects. The technique and the identified significant gene-trait pairs could be of broad interest to the field. To be published in Genome Biology, I only have three major points as shown below:

1. While the authors compared the performance of several strategies using stimulated data, it would be great if the comparison could be expanded to real data, with other newly published methods (i.e. FOCUS) included.

*We appreciate this comment. In agreement with the reviewer, we have now added a new analysis to systematically compare the PTWAS scan procedure with different available methods, including SMR, Fusion, and PrediXcan, using the real data. In particular, we select the height data from the GIANT consortium (in forms of single-SNP z-scores) and the whole blood eQTL data from the GTEx (v8). We follow the instructions in the corresponding original papers to construct the eQTL weights for each method in comparison. To perform association testing, we use the software package GAMBIT, which implements a generalized burden test utilizing summary-level statistics of GWAS. This decision ensures the comparisons are focused on the constructions of (composite) instrumental variables.*

*In newly added Table 1, we report the significant genes identified by different methods at the 5% level for the FDR control and the Bonferroni correction procedures. In brief, we find our results from this real data analysis markedly consistent with what we observe in the simulation studies. Specifically, we find that methods accounting for allelic heterogeneity empirically outperform the single-SNP based SMR approach, and the proposed PTWAS scan approach yields the highest number of discoveries.*

*In the revised manuscript, we have added a paragraph on page 8 to describe this new analysis and its results. The data used in this analysis are also made available at https://tinyurl.com/ wg8p72k for reproducibility purpose.*

*We have also carefully considered the reviewer’s suggestion of comparing with recently proposed method, FOCUS. In the end, we conclude that the main feature of FOCUS, as well as newly proposed method TWMR, is to tackle the identifiability issue originated from correlated com­posite IVs (which are constructed from correlated gene expression data). We acknowledge that this is a critical issue for the TWAS analysis. On the other hand, the composite IV construction at the single gene-level by FOCUS is not different from the TWAS-Fusion. In fact, the single gene-level association z-statistics derived from the TWAS-Fusion are used as the input for the FOCUS (see Equation 1 of the Results section and the description of dataset in [1]). To focus on evaluating the strengths of the composite IVs constructed from single genes, we decide not to compare with FOCUS or TWMR in the current paper. Nevertheless, we have made these points clear in the Discussion section of the revised manuscript, and we will consider integrate PTWAS into the framework of FOCUS in our future follow-up work.*

2	The paper re-discovered several known gene-trait relationships (with citations missing). How­ever, more evidence are needed for validating whether the result is biological meaningful (i.e. the intersection with OMIM database or other approaches as shown in the TWMR paper: https: // www. ncbi. nlm. nih. gov/ pubmed/ 31341166

*We greatly appreciate this suggestion. Following the reviewer’s advice, we have added a new analysis to examine the enrichment of the heart-disease PTWAS significant genes (which pass the causal model diagnosis) in the relevant OMIM terms. Specifically, we select a subset of high confidence gene-trait associations constructed in our PhenomeXcan paper [2], with an exclusive focus on heart disease. The construction of the high confidence gene-trait associations from the OMIM is described in detail in [2]. We then overlap the PTWAS significant genes from 4 heart disease-related traits, including CARDioGRAM CAD, UKB diagnosed heart disease, UKB self-reported hypertension, and HRgene heart rate study). The overlapping is assessed to be highly significant (p-value = 5.7 × 10−10 by Fisher’s exact test). The details of this analysis are given in the third paragraph of page 9 in the revised manuscript. In the newly added supplementary table S3, we provide the detailed information of overlapping between the PTWAS significant genes and the OMIM terms.*

3	The related github website needs more detailed tutorials (i.e. installation, parameters) for broad application of the computation strategy. For reference, please check the tutorials of the PediX-can (https: // github. com/ hakyimlab/ PrediXcan/ tree/ master/ Software ) or MetaXcan (https: // github. com/ hakyimlab/ MetaXcan ). Also it would be great if the computation time and resource could be briefly annotated in the method section.

*We thank the reviewer for this comment. Following the reviewer’s suggestions, we have now provided more details on the compiling, installation, and usage of the software. We are committed to continuously improving the Github site. We also provide the references to computational time in TWAS scan and estimation in the Method section, as suggested.*

Reviewer 2

The paper proposes PTWAS, a computational framework to detect association and investigate causal relationships between gene expression and complex traits. The proposed method tries to make use of the established concept and principles from instrumental variables (IV) analysis to claim causal relationships. The idea is interesting and has the potential to play an important role in understanding the complex causal mechanism of a biological system. However, I have the following concerns about the current form of the paper.

1. The logic flow and the workflow of the proposed method need to be better demonstrated and explained. The proposed method is a complicated data processing procedure involving multiple inputs and many steps. The paper, however, does not put together all these ingredients into a well-organized system whose logic can be easily followed. The workflow showed in Figure 1 is somehow too ”high-level” with many key components and key steps missing, and thus does not help much for understanding the proposed approach in depth. It is not clear to me what are the inputs (in a concrete form) of the proposed approach and what are the intermediates obtained during the learning process. I hope the authors can better the workflow and highlight the inputs, key intermediates and final outputs in a concrete form.

*We appreciate the reviewer’s comment and agree with this assessment. We have entirely re-made Figure 1 (the workflow chart) to include the information on input, key intermediates, and final output, as suggested by the reviewer.*

2. It’s not transparent how the causal assumptions are verified. Verifying the causal assumptions is a critical step for the proposed method. Based on the statement of the paper, this task heavily depends on the “two-sample design”. However, the paper talks very little about the design, how data are used based on the design, how and why such a design can help establish a proper IV, leaving a significant gap in understanding.

*We thank the reviewer for this comment. In retrospect, we find that our original writing does not provide sufficient details on the promise and the limitations of the proposed model validation procedure, neither does it provide sufficient background and intuition on the topic.*

*Following the reviewer’s suggestion, we have now re-written the relevant sections, especially the subsection of ”Validating causal assumption” in the section of ”Method overview” (Page 4), to provide more references on the rationale and the intuition of the proposed validation procedure. We have also made explicit connections to the existing diagnosis approaches and explained their distinctions in the context of PTWAS analysis. Finally, we believe the proposed validation procedure, in principle, is not restricted by the settings of the two-sample design [3] (although we only consider its application in the two-sample design setting for this paper).*

3. Some notations in the paper are not explicitly defined or explained. For example, the concrete meanings of in P12-L60 and in P13-L7 are missing; in page 13 are not defined. These vague notations and concepts result in confusions and obstacles in reading and understanding.

*We thank the reviewer for this comment. We have re-written the relevant sections to clarify the relevant concepts, abbreviations, and notations.*

4. Result validation and comparison. The paper compares PTWS to competing methods such as PrediXcan, TWAS-Fusion, SMR via simulation, and compares PTWS to colocalization analysis on real data. I am wondering whether PTWS can also be compared to PrediXcan, TWAS-Fusion,SMR on real data. Moreover, the paper chooses the ”colocalization probability” as the standard for result validation. Why colocalization probability is a proper choice? Do we have some other measurements can serve better for the validation purpose?

*We appreciate this comment from the reviewer. We have now performed additional analyses to compare the empirical power of the PTWAS scan procedure to the existing methods mentioned by the reviewer. Please refer to our responses to the first two comments by Reviewer 1 for more details.*

*With respect to the comparison with the colocalization analysis, our intention is NOT to regard the colocalization analysis as a validation of the TWAS analysis, and we apologize for the confusion. Instead, we aim to show the relationships between these two distinct types of integrative genetic association analysis approaches in a real data example. In the revised manuscript (Page 10), we have explicitly clarified that colocalization analysis is a different type of integra­tive analysis approach that serves complementary goals as the TWAS analysis. Specifically, it aims to identify the overlapping of GWAS and eQTL signals at the single-variant level. We also acknowledged that from the theory of the IV analysis, the colocalization of the causal variants is not a necessary condition to imply the casual relationships from genes to traits. Nevertheless, we suspect that confidently identified colocalization signals also tend to drive the TWAS scan signals. Our observation in the manuscript seemingly fits such expectations. Through this anal­ysis, we hope to convince the readers that the two types of integrative genetic analysis can yield correlated results and complementary to each other.*

*References*

*[1] Mancuso, N. et al. Probabilistic fine-mapping of transcriptome-wide association studies. Nature genetics 51, 675 (2019).*

*Pividori, M. et al. Phenomexcan: Mapping the genome to the phenome through the tran-scriptome. BioRxiv 833210 (2019).*

*Burgess, S., Bowden, J., Fall, T., Ingelsson, E. & Thompson, S. G. Sensitivity analyses for robust causal inference from mendelian randomization analyses with multiple genetic variants. Epidemiology (Cambridge, Mass.) 28, 30 (2017).*

**Second round of review**

**Reviewer 1**

The authors have satisfactorily responded to most comments. In particular, the additional details concerning biological validation, performance on real data and the updated website are appreciated. Overall, I think this manuscript should be read by a wide audience.

**Reviewer 2**

The authors answered my questions well. I have no further concerns.
